# The inTouch Integrated Care Framework – Reimagining Integrated Health Service Delivery

**DOI:** 10.5334/ijic.8638

**Published:** 2025-08-29

**Authors:** Kathy Eljiz, Jo Medlin, Ben Harris-Roxas, Jasmin Ellis, Alison Derrett, Graeme Loy, David Greenfield

**Affiliations:** 1 Health Services Research Unit, School of Population Health, University of New South Wales, AU; 2 Research and Education Network, Western Sydney Local Health District, AU; 3 Integrated and Community Health, Western Sydney Local Health District, AU; 4 School of Population Health, University of New South Wales, AU; 5 Western Sydney Local Health District, AU

**Keywords:** integrated care framework, systems approach, community, acute, primary health, aged care

## Abstract

**Introduction:**

Integrated care has been adopted as a guiding principle to reduce fragmentation and to make health systems more person-centred. Successful integration requires care services and processes that include primary care, specialist care, and acute services. To promote learning and development in the field, flexible and adaptable empirically derived frameworks for different contexts, conditions and settings are required.

**Description:**

The Western Sydney Local Health District (WSLHD) inTouch Program is a systems approach to reconceptualising service delivery, focusing on integrating systems components, platforms for service delivery and coordinating care planning for improved care delivery. The inTouch Program key features and core elements, and three care pathways are presented, demonstrating the adaptability for different population cohorts.

**Discussion:**

The inTouch Program, and each pathway, embodies an ongoing, dynamic response to patient priorities. Informed by bottom-up stakeholder input from staff, consumers, and care providers, each pathway fosters interorganisational collaboration by facilitating coproduction of care across primary, aged, and community care sectors, as well as hospitals. The analysis derives an inTouch Integrated Care Framework consisting of six core elements, four strategy and governance enablers, and operational components required for practical implementation and sustainability of pathways. Together they operationalise and link diverse health and social care organisations into a living continuum of care.

**Conclusion:**

Accounting for nuances in organisational and care settings, is the difference between success and failure for integrated care. The exploration of the inTouch program and three pathways has detailed how to deliver care for different patient cohorts across health and social care settings. The three care pathways demonstrate the viability and necessity to pursue collaboration, cooperation and care navigation, whilst tailoring services to specific contexts and stakeholder needs. inTouch program aims to ensure safe, high quality patient care is delivered across health and social settings, prioritising the patient needs.

## Introduction

Globally, integrated care has been adopted as a guiding principle to reduce fragmentation and to make health systems more person-centred [1]. Integration has long been viewed as a solution to fragmentation of care, increased complexity in delivery of care, and poor quality of care [2]. However, integrated care is not a simple, “one size fits all” approach: care means different things to different people [3]. The differences are highlighted by the ongoing debates about what exactly constitutes integrated care [4]. Regardless of the definition of integrated care adopted, there is understanding that it strengthens health services by being flexible, people-centred [5], multi-morbidity focused [6] and deliverable by multi-disciplinary teams [7] across different settings. With a rise in chronic conditions across OECD counties [8], integrated health systems focus on delivering appropriate care, preventing people from being in the acute system unnecessarily. This goal requires integrated care services and processes that include primary care, specialist care, and acute services [9,10].

In many countries there is an increasing recognition across health and social care providers and commissioners that care needs to focus on supporting people to remain well at all stages of life. Key strategies to achieve this are supporting people to receive care in community settings close to home [11], and developing integrated care systems that encompass health and social services [12], such as housing, welfare and education. We know that attaining integration of services across and within different settings, organisations, and professions enhances value in service delivery and reduces silos [13]. However, achieving a shared vision and common understanding of what integration looks like across multiple services, health and social care professionals, and patients, is a significant challenge [14,15]. Furthermore, it can be difficult to ensure the seamless flow of information between service providers across different settings [16], in a timely and contextually sensitive way [17]. The lack of agreement around integration extends to a lack of clarity about how to achieve it, including how more integrated care can be supported and facilitated. If integrated care is to be achieved change is required to promote primary and acute care planning together to break down silos in health service delivery [14,18]. That is, a complete paradigm shift so that care planning considers health, welfare and social circumstances in determining health and community services.

Whilst frameworks for integration exist, these tend to concentrate on meeting the needs of targeted groups and populations with defined chronic diseases [19]. Integrated care framework efforts tend to be on integrating care vertically or horizontally but not both [4], with calls for more comprehensive models that address multi-morbidities [20]. In response to this, maturity models and system appraisal frameworks for integrated care have been developed, such as the SCaling IntegRated Care in COntext (SCIROCCO) project that focuses on the facilitating factors and pitfalls of integrated care initiatives in five European regions [21], and the Project INTEGRATE framework to assess people-centred integrated care processes [22] developed using lessons from literature. At a systems level, governments are formulating polices and promoting frameworks to achieve the integration of care, for example the NSW Health Integrated Care Strategic Framework [23]. Other frameworks cover different settings such as: the integrated behavioral health (IBH) Cross-Model Framework aimed at primary care clinics who are trying to use and sustain integrated behavioral health [24]; the ICP OP 10 Step Framework, a framework to implementing integrated care for older persons [25]; and the Navian Hawaii’s Integrated Care Program, separate to their Hospice Care team, working with those who are seeking curative treatment [26]. What is common across these frameworks is that successful integration of health and social care depends on the implementation of initiatives that are locally focused [27,28] and secure meaningful engagement of staff, consumers and care providers [28,29].

Successful integration of health and social care depends on the implementation of initiatives that are based on local needs and conditions [27]. They have been implemented using a bottom-up approach and secure meaningful engagement of staff, consumers and care providers [28,29]. To improve health system efficiency, we require integrated care frameworks that are flexible and adaptable for different community contexts, conditions and settings. Individual patient care needs must be balanced with the broader social and care needs of a community in line with a population or public health perspective [30]. In working towards developing and sustaining effective, safe health care systems, we need to deliver care in the community with access to specialist services that do not require an admission, or readmission, into acute services [31].

Western Sydney Local Health District (WSLHD) initiated and has facilitated the development of the inTouch Program, linking services within its geographic region. inTouch is an integrated care strategy for safe, high quality patient care delivered in the appropriate environment. The inTouch Program provides an innovative approach to integrate services by facilitating cooperation and care navigation between providers across primary, aged, community and acute settings. Using a case study approach, we focus on WSLHD, describing and analysing the inTouch Program and its three current pathways to present the empirically derived inTouch Integrated Care Framework.

## The inTouch Program

### Setting

The setting for inTouch is WSLHD (the District). WSLHD provides public healthcare across more than 120 suburbs spanning 780 square kilometres. With a population of over one million people, WSLHD is the fastest growing region in New South Wales (NSW), Australia with a projected population of 1.3 million residents by 2031. The District services a diverse population with 50% born overseas, 54% speak a language other than English at home and is home to the largest Aboriginal and Torres Strait Islander population in NSW. WSLHD employs over 13,000 staff across 70 sites, delivering acute, integrated care and community-based services [32].

### Purpose, key features and core elements of the inTouch Program

The integrated care strategy at WSLHD began in 2006 with the goal of developing clinical pathways or models of care for specific chronic disease conditions. In NSW, there have been State level policy directives with local health districts being supported to implement initiatives that meet the needs of the population [33]. For WSLHD, the catalyst was in response to increasing demand for disease management of specific chronic conditions. They required a more effective whole of health service response. Across the 17-year period and multiple iterations, the WSLHD integrated care strategy evolved by using a bottom-up approach, responding to local level needs by engaging with staff, care providers, current patients and carers, and consumers that have used or may use health services.

The inTouch Program [34,35,36] was designed and implemented by WSLHD across 2021–2023. The Program is an integrated care strategy that provides community-based care to people who are at risk of avoidable emergency department attendance or hospitalisation. Investment into inTouch is substantial with resources committed to the Program via new models of care necessitating dedicated staff, operational equipment and procedures. Care is delivered by enhancing access to appropriate community-based services including general practice, community health, private allied health, and specialist outpatient care. The goal of the inTouch Program is to develop a safe, high quality, patient centred system of community-based health services to assist with the management of chronic conditions and multi-morbidity, supported by the expertise within acute services. The Program’s success has been recognised at the NSW State level being nominated as a finalist in the NSW Premier’s Award in the category of Highest Quality Healthcare [37] and featuring as a case study in the State of the NSW Public Sector Report 2023 [38].

The inTouch Program has six core elements, which guide the development and implementation of pathways through defining a clear focus and then integrated process and actions (Table 1). Currently, the inTouch Program has three pathways which are the means by which the care management processes and requirements, such as assessments, models of care and resources, needed to provide long term care for defined groups of people [39] are delivered. The inTouch Program elements can be used to address diverse patient health and social needs, including morbidities through the development of additional pathways.

**Table 1:** inTouch core elements, focus, processes and actions.


ELEMENT	FOCUS	PROCESSES AND ACTIONS

1. Stakeholder engagement and risk analysis	Identification and engagement of people who are at risk of hospitalisation	***Targeted enrolment:*** health professionals nominate patient/ client for assessment of patient’s chronic risk - Enrolment of individual (>16 years) with a primary diagnosis: – Diabetes, – Chronic Obstructive Pulmonary Disease (COPD), – Chronic Heart Failure (CHF), – Coronary Artery Disease (CAD), or – Hypertension. Patient, based on their hospital admissions over the most recent 12-month period, classified – – ***‘Very High Risk’ category*** with >3 ED presentations or unscheduled admissions. – ***‘High Risk’ category*** >1 ED presentations or unscheduled admissions.

2. Population and individual care plans	Care plans developed to address population and individual health needs and risk factors	***Comprehensive assessment:*** standardised, purpose designed tools used to develop a Shared Care Plan (SCP) enabling continuity of care; multiple domains assessed including medical, functional (physical), psychological, social and physical factors. Actions include: identify best practice care for the mobility of the patient, addresses individual care needs beyond disease management and treatment, focusing upon psychosocial care needs, e.g. an individual’s living and financial needs or using interpreters for members of the CALD community. sharing assessment information via the Connecting Care Contact Centre (CCCC) also known as the single point of access (SPA). A SCP is - developed in partnership with the patient and GP with specialist input; informed by a Comprehensive Assessment, GP Management Plan and/or a Discharge Plan; based on a Medicare Team Care Arrangement (TCA); the basis for scheduled monitoring and review utilising Medicare Benefits Schedule (MBS) items for case conferencing and Team Care Arrangements (TCA) review; clearly identifying clinicians and responsibilities within a multi-disciplinary team; and, clearly nominating a care coordinator/case manager.

3. Continuum care coordination	Connecting patients to services across the continuum: acute, ambulatory, general practice and community health through navigation or referral to services	Three levels of integrated care delivery – linkage to services, care coordination or case management – provided for clients. Coordinated cross service discharging-engagement processes and information sharing. Care management GP responsibility when a community care option is organised.

4. Shared decision making	Shared decision making between the patient, carer and health care providers	Patient informed decision-making central principle. Patients are given care options including from GPs, specialists and other health professionals. Care coordinators explain SCP, advise actions and decisions for patient, documenting choices and ramifications.

5. Knowledge translation and health literacy	Bridge the patient knowledge gaps in health information and services to be able to make appropriate care decisions	Individuals are given self-management telephonic health coaching to improve health literacy and capacity to manage their disease. Interventions include face-to–face discussions, group programs, and a chronic condition rehabilitation program. Establishment of a patient self-management action plan and goals incorporated into the SCP.

6. Monitoring, review and adaptation	Identification of disease exacerbation, psychosocial issues and the need for initiation of early service provision	***Patient level*** individual care plans are continuously monitored, reviewed, adapted and implemented, ongoing iterative cycle with clinicians, patients and carers. Clinical service delivery review simultaneously with SCP review and, where possible, the Medicare Benefit Scheme items review. ***Program level*** aggregation, monitoring and evaluation of data to determine impact and outcomes - Data reviewing – inbound referrals, patient acceptability (eligibility/exclusion), SCPs, care coordination and self-management support, service utilisation a, unmet needs and patient experience and satisfaction. Evaluation of resource utilisation assessment linked to outcomes measures. Patient, carer, and consumer feedback including verbal and written.


### inTouch Pathways

WSLHD partnered with multiple healthcare providers to develop and deliver three inTouch pathways. External healthcare providers included NSW Ambulance, Western Sydney Primary Health Network (WSPHN), general practitioners (GP), and 65 aged care residential facilities. This Program is delivered in the inTouch operational service that operates through a central point of contact, a call service functioning from 8 am to 8 pm 7 days per week. The phone line is a free number that serves as a single point of access for all three inTouch pathways. The phone line is staffed by a triage clinician supported with a procedure manual and clinical guidelines that inform responses to inquiries. The triage clinician is qualified to provide preliminary advice regarding immediate and follow-up care. Appointments for further care can be organised for services within the District or with GPs.

The three inTouch care pathways are: 1. inTouch COVID Care in the Community [40]; 2. inTouch Residential Aged Care Facilities (RACF) [34]; and, 3. inTouch Planned Care for Better Health (PCBH) [41]. A summary of the three care pathways is discussed and presented below (Table 2), demonstrating that the elements can be adapted across different settings, contexts and conditions.

**Table 2:** Overview of three care pathways of the inTouch Program.


1. inTouch COVID CARE IN THE COMMUNITY	

**Lifespan**	January 2021 – ongoing

**Purpose/aim**	Care for COVID-19 patients: not requiring face-to-face clinical care or intervention; and early identification and intervention when deteriorating.

**Partners**	WSLHD Specialty teams NSW Ambulance NSW Health COVID response

**Evaluation/monitoring**	NSW Ambulance and WSLHD COVID Monitoring Framework WSLHD inTouch COVID Clinical Governance Framework

**Data sources**	WSLHD NSW Ambulance NSW Health NSW Police

**Actions**	COVID-19 patients initial assessment of symptoms, medical and social risk factors – assigned a risk category – Low or High Patients receive follow-up assessment calls based on their risk category; if risk profile changes reclassification of category. inTouch clinicians provide resources and information to support the wellbeing of patients, including how to access additional community services. Patients self-manage COVID-19 symptoms and actions if deteriorating/ improving.

**Activity**	Data reported January 2021-November 2023: 47,587 patients have received care on pathway, 16,485 cared for directly by WSLHD 31,767 were cared for by Calvary/Medibank under contract with WSLHD using the pathway. Impact: During the peak of the pandemic in 2021 WSLHD inTouch - admission rates were 4.6% compared to 25% NSW, and death rates were 0.04% compared to 1.4% for NSW.

**2. inTouch RACF**	

**Lifespan**	April 2022 – ongoing

**Purpose/aim**	Improve aged care residents’ health and wellbeing through person-centred, flexible, timely care

**Partners**	Aged care residential facilities (n = 65) NSW Ambulance WSLHD Specialty teams Primary Care through WSPHN

**Evaluation/monitoring**	NSW Health Urgent Care Service Evaluation Framework NSW Ambulance and WSLHD RACF Monitoring Framework Analysis of WSLHD activity & hospital utilisation data sets

**Data sources**	WSLHD NSW Ambulance

**Actions**	Pathway provides: Specific referrals process for NSW Ambulance, general practices, and RACFs; Continuous monitoring of the NSW Ambulance Arrivals Board to identify suitable patients; Virtual clinical assessments and triage to identify appropriate ED alternative care plans; SCPs developed in partnership with patient/carers, speciality medical teams, GPs, RACF providers and other community-based services; and, Referrals and care navigation to services delivered in residence, including mobile diagnostics capability for rapid assessment and treatment.

**Activity**	Data reported April 2022-November 2023: 5,127 referrals received. Access savings include: 1,965 avoided (38%) transfers of residents to EDs. 11% reduction in readmission (based on the usual rate within 28 days). 2% reduction in ED representations (based on the usual rate within 48 hrs). NSW Ambulance savings: 2,692 paramedic hours (based on average of 101m of case cycle time). NSW Patient Transport Service savings: 1,562 return trips to RACFs.

**3. inTouch PCBH**	

**Lifespan**	July 2022 – ongoing

**Purpose/aim**	Facilitate and strengthen care for patients identified of being at risk of unplanned hospitalisation within 12 months.

**Partners**	Primary health network (PHN) WSLHD Specialty Teams Justice Health

**Evaluation/monitoring**	NSW Health Integrated care Monitoring and Evaluation Framework

**Data sources**	NSW Health: Integrated Care Outcomes Database (ICOD) Patient Flow portal WSLHD activity and hospital utilisation data sets NSW Health Integrated Care Outcomes Database

**Actions**	Mutli-disciplinary shared care model: Care Facilitators undertake coordinate care and navigate access to services, and health coaching, General practitioners and acute care specialty teams deliver clinical care.

**Activity**	Data reported July 2022–November 2023: 8,097 referrals received. Post hospital discharge services delivered include: 5,053 calls to people who are at high-risk of hospitalisation addressing medication management, additional medical (re)tests and/or referral/ follow-up with GP; 840 calls by Aboriginal Health Practitioner to Aboriginal people with chronic conditions; 58 Type 2 Diabetes multidisciplinary case conferences; 1,994 episodes of supportive post discharge transfer of care to GPs and/or self-management support to people with Atrial Fibrillation; 316 referrals for care navigation to inTouch referral support team; and, 136 referrals to COACH program team for lifestyle risk factor modification health coaching.


#### inTouch Pathway 1 – COVID Care in the Community

**Figure 1:**
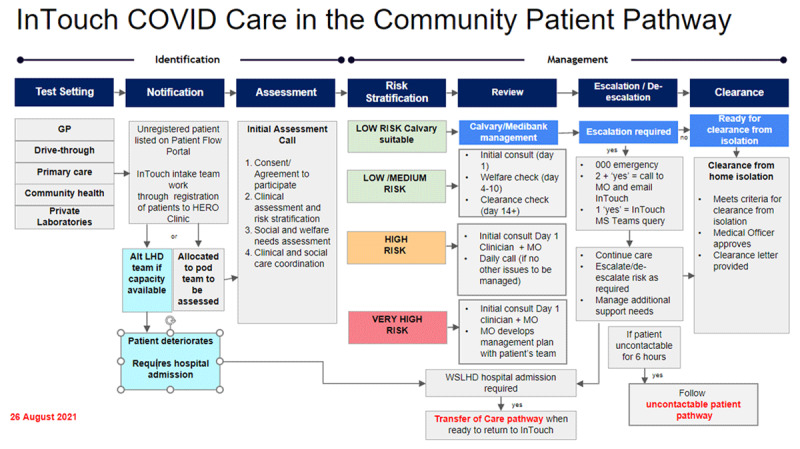
COVID Care in the Community.

inTouch COVID Care in the Community was developed January 2021 in response to the COVID-19 Delta outbreak. inTouch COVID Care in the Community proactively manages and monitors COVID-19 positive patients who do not need face-to-face clinical care and provides early identification and intervention to COVID-19 positive patients who are deteriorating (Figure 1; Table 3).

**Table 3:** inTouch Pathway 1 – COVID Care in the Community.


ELEMENT	APPLICATION

**1. Stakeholder engagement and risk analysis**	This pathway reduces unnecessary emergency department and hospital admissions by: identifying and rectifying deterioration early in COVID-19 positive patients; and, supporting patients to self-manage at home.

**2. Population and individual care plans**	The initial assessment checklist addresses: consent to engage with inTouch; a risk profile evaluation; symptom identification and clinical history; mental wellbeing screening; and, health literacy review. A SCP is established using a template with options to refer to WSLHD services and private primary health and welfare services in the community.

**3. Continuum care coordination**	Support and care coordination for patients as required, including: clinical care provided by Hospital in the Home (HITH); mental health services; and/or, welfare and social care services.

**4. Shared decision making**	Adult Short Screening Questionnaire administered to patients to: determine pathway suitability; decision-consent capabilities; identification of risk/ high-risk clinical indicators; and, required education and resources.

**5. Knowledge translation and health literacy**	Health literacy resourcing: patients given health information and skills education regarding physical, mental, and social health needs.

**6. Monitoring, review and adaptation**	Ongoing SCP assessment addressing: physical health and COVID-19 symptom assessment; – mental wellbeing screening; consent and health literacy; and, care plan, including services needed and escalation strategy.


#### inTouch Pathway 2 – Residential Aged Care Facilities (RACF)

**Figure 2:**
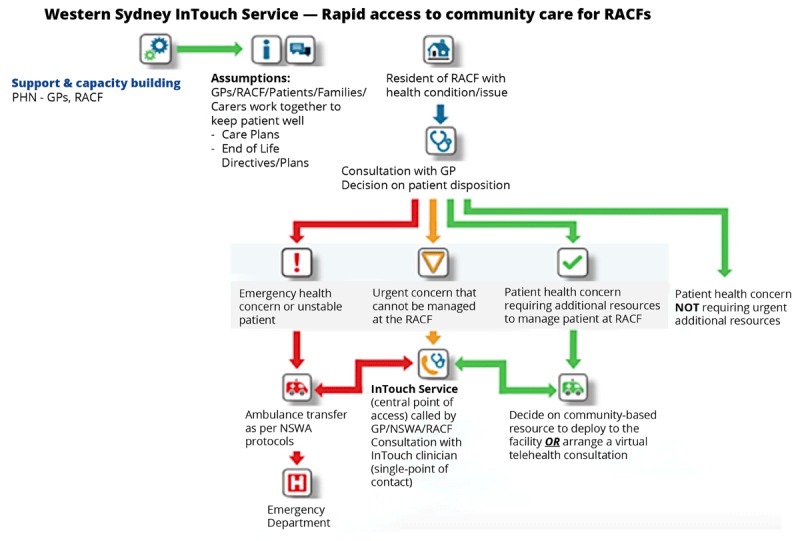
inTouch Residential Aged Care Pathway.

The inTouch Residential Aged Care Service (RACS) pathway was established in April 2022. The pathway provides a single encounter of care for ‘primary care type’ low acuity conditions such as falls and wound care of aged care residents to prevent ED presentations and increase a resident’s options of care setting (Figure 2; Table 4).

**Table 4:** inTouch Pathway 2 – Residential Aged Care Facilities (RACF).


ELEMENT	APPLICATION

**1. Stakeholder engagement and risk analysis**	The inTouch RACS pathway criteria is people who reside in a aged care facility (n = 65) within the WSLHD geographical boundary. The pathway increases options for the delivery of primary care needs without a resident having to leave their facility. Service stakeholders include GPs, RACF clinicians, NSW Ambulance, Paramedics, and the Virtual Clinical Care Centre (VCCC).

**2. Population and individual care plans**	The initial assessment checklist addresses: consent to engage with inTouch; a risk profile evaluation; symptom identification and clinical history; mental wellbeing screening; and, health literacy review. A SCP is established using a template with options to refrfer to WSLHD services and private primary health and welfare services in the community.

**3. Continuum care coordination**	inTouch RACS pathway ongoing care coordination involves determining the service best placed to do so – either inTouch clinician, GP or Primary Centred Medical Home. Within care coordination is the provision of urgent responsive community care alternatives for low acuity conditions.

**4. Shared decision making**	Shared decision making enabled via: patients and professionals co-design care plan – participate in identifying needs, options and decisions re health and social issues; case conferences and information sharing with acute care, community and primary health services.

**5. Knowledge translation and health literacy**	Health literacy resourcing: health professionals, carers and patients given health information and skills education regarding physical, mental, and social health needs, and services – acute, primary care and community – to address them.

**6. Monitoring, review and adaptation**	Ongoing SCP assessment addressing: physical health-illness and symptom assessment; mental wellbeing screening; consent and health literacy; and, care plan, including services needed and escalation strategy. Program level evaluation incorporates monitoring of ED presentation rates and hospital utilisation by individuals from RACF.


#### inTouch Pathway 3 – InTouch Planned Care for Better Health (PCBH)

**Figure 3:**
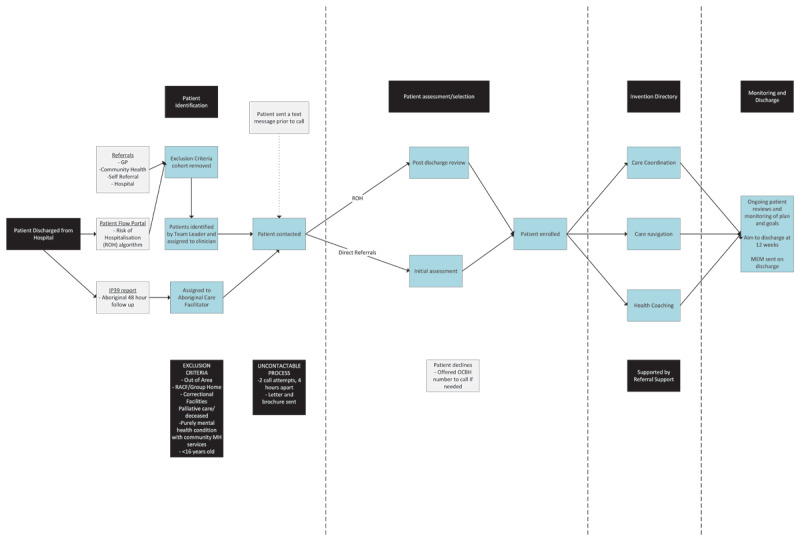
inTouch Planned Care for Better Health Pathway.

The inTouch PCBH service has been operational since July 2022 and was developed using the inTouch elements in response to one of the eight NSW Health NSW Integrated Care Initiatives, Planned Care for Better Health [42] (Figure 3; Table 5). The PCBH pathway improves the patient’s experience of care and keeps patients healthier over the long term to ultimately prevent ED presentations and potentially preventable hospitalisations. Many of these patients will have complex and chronic needs.

**Table 5:** inTouch Pathway 3 – inTouch Planned Care for Better Health (PCBH).


ELEMENT	APPLICATION

**1. Stakeholder engagement and risk analysis**	The Risk of Hospitalisation (ROH) algorithm (37) is used plan patient centered interventions. The pathway criteria is patients identified as being at-risk of unplanned hospitalisation in the next 12 months and an ongoing condition:diabetes, asthma, renal, COPD/respiratory, cardiac; palliative (but services not set up); oncology (in respite but not palliative); mental health; dialysis; gynecology; or oncology.

**2. Population and individual care plans**	Care planning involves assessing patient’s hospitalisation and medical history, demographic and socioeconomic factors. Then enrolling patient into a 12-week program with an intervention – care navigation, care coordination and/or health coaching. The PROMs measure, PROMIS-29, is used to assess intervention effectiveness.

**3. Continuum care coordination**	Coordination of patient-centred care role involves providing timely access to community health services by initiating referrals, removing barriers to access, and liaising with assessment teams and service providers.

**4. Shared decision making**	Shared decision making enabled via: patients and professionals co-design care plan – participate in identifying needs, options and decisions re health and social issues; case conferences and information sharing with acute care, community and primary health services.

**5. Knowledge translation and health literacy**	Health literacy resourcing: health professionals, carers and patients given health information and skills education regarding physical, mental, and social health needs, and services – acute, primary care and community – to address them. Education of patient self-management support techniques to improve clinician-patient communication, increase patient self-efficacy, health literacy and promote consumer enablement. These include motivational interviewing, teach-back and action plans.

**6. Monitoring, review and adaptation**	Ongoing SCP assessment addressing: physical health-illness and symptom assessment; mental wellbeing screening; consent and health literacy; and, care plan, including services needed, escalation strategy, and discharge plan and timing (>12 weeks).


## Discussion

The study has described and analysed inTouch Program and its three current pathways. Driven by both policy imperatives and local service demands, each inTouch pathway embodies an ongoing, dynamic response to patient priorities. Informed by bottom-up stakeholder input from staff, consumers, and care providers [31,32], each pathway fosters interorganisational collaboration by facilitating coproduction of care across primary, aged, and community care sectors, as well as hospitals [20,29,30]. This patient-centered approach challenges traditional care silos and boundaries [20] by building interdisciplinary teams, with strong primary care involvement, that address individual needs beyond clinical care, encompassing broader social determinants of health [14,34]. The outcome is the development of robust, high-quality integrated care program and pathways spanning across health and social care boundaries [33].

Analysing the application of the six core elements across three care pathways, the inTouch Program offers lessons for replication in diverse settings. The three key lessons of the inTouch Program are: (i) a structured process to support service redesign; (ii) a single point of contact for services, with streamlined clinical processes to facilitate efficient access to general practice, community health, allied health and specialist outpatient services; and, (iii) service innovations in digital health and patient self-management. The focus of inTouch is directed to integrating strategic management factors (including governance, culture, partnerships, service providers and users and patient outcomes), platforms for service delivery (face-to-face and virtual care including telehealth) and the most appropriate environment for care delivery (acute, outpatient clinics, home and community care providers).

**Figure 4:**
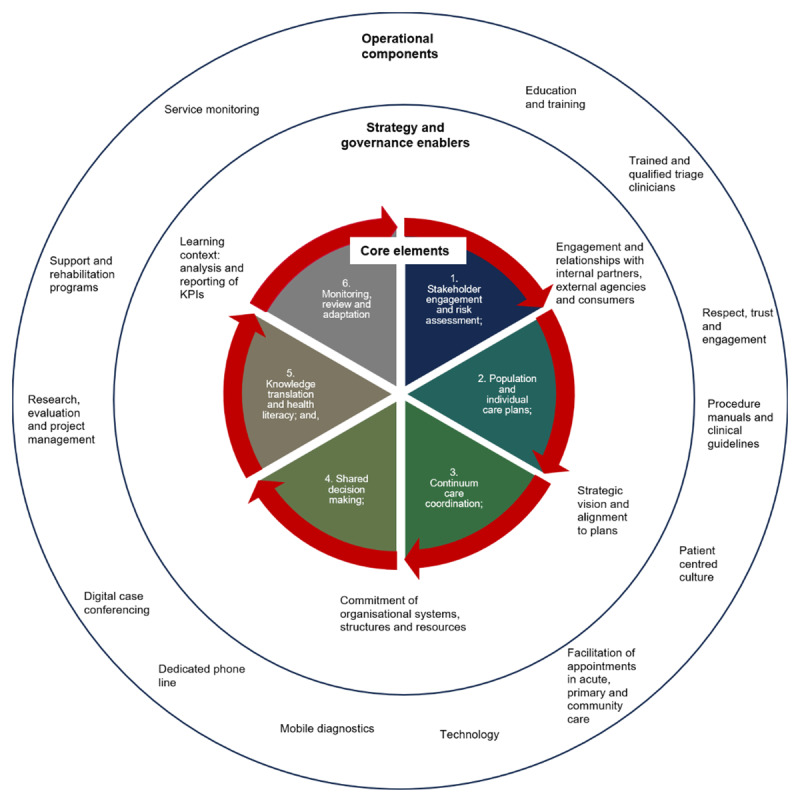
InTouch Integrated Care Framework.

From the analysis of the three pathways emerges an empirically grounded framework to direct integrated care actions and system learning (Figure 4). The centre of the model is the six core elements of inTouch that guide the development of pathways (Layer 1). The middle component is the four strategy and governance enablers, which translate the core elements into pathways (Layer 2). The outer ring is the operational components required for practical implementation and sustainability of pathways (Layer 3). This cohesive, comprehensive model addresses the intrinsic complexities associated with a pragmatic, multi-morbidity approach to integrated care [28].

The four strategic and governance enablers translate the core elements into service activities, patient and professional engagement actions and systems learnings.

The first enabler is undertaking active engagement and ongoing nurturing of relationships with internal services, external partners and consumer groups in the integrated care initiative. Stakeholder engagement and ownership is required to achieve commitment and success of integrated care initiatives [43]. Specifically, this is about creating an environment that promotes interorganisational collaboration by facilitating coproduction of care across the health sector including primary, aged and acute, and community services. Connecting stakeholders, including patients and professionals across health and social services, promotes transformational integration through meaningful engagement [44]. Stakeholder’s different perspectives and roles are harnessed to work towards a common purpose of ensuring high quality, patient centered care along the care continuum. Integrated care, particularly for chronic disease management and population health priorities, necessitates the engagement of multiple providers [45]. Clinical engagement, particularly doctors [46], in the planning, implementation and evaluation of integrated care initiatives is fundamental. Adopting the “tight-loose-tight” approach stakeholders have flexibility in the development and implementation of each unique pathway to achieve their common goal [47].

The second enabler is strategic vision and alignment with clear links to policy and organisational level goals, plans and actions. Senior professionals promote the understanding that integrated care is a long-term endeavour, requiring sustained vision and ongoing perseverance. In the NSW context, the Strategic Framework for Integrating Care [23] and the NSW integrated key initiative of Planned Care for Better Health [42] provide strategic guidance for integrated care across the State. The WSLHD Strategic Priorities [48] also shapes the ongoing development of the inTouch Program thereby providing clear alignment with health, social and community care priorities, programs and partner organisations. Different health and social care services share a common goal, whilst adhering to independent organisational boundaries and governance structures [1], and patient privacy regulations [49].

The third enabler is the commitment of organisational systems, structures and resources necessary for integrated care – across service, management and executive levels. Successful integrated care pathways require long term strategic planning, with the commitment of multidisciplinary staff and the investment of dedicated resources [50] to address fragmented resource allocation [51]. As the coordinator of inTouch, WSLHD uses a purpose-designed operational manual, with dedicated, trained staff to ensure standardised assessment, planning and coordination of care. This promotes communication and integration, and reduces the likelihood of fragmentation in service provision [15]. The dedication of protected resources on an ongoing basis rather than ad hoc funding signals to organisational members, external agencies and patients that the initiative is part of the long-term fabric of the organisation.

The fourth enabler is creating a learning context. The requirement is for access to timely information and staff with the abilities to analyse and produce reports on defined key performance indicators for stakeholders. Doing so reinforces commitment, shared goals, ongoing information sharing and collaborative problem solving – at both the program and pathway levels – enacting evidenced based decision making [52]. Information and communication technologies are necessary tools to achieve a collaborative, dispersed learning ecosystem [12]. At the individual patient level, cross organisational and service care plans are the mechanism to record and coordinate actions and responsibilities. Care plans make decisions transparent, enabling patients to be proactive managers of their health [53]. The plans allow patients and carers to provide concurrent feedback to further improve care at the individual patient and Program levels. When integrated and working together these components create a learning health system which improves practices, process and ongoing collaborative learning at patient, service and organisational levels [54].

There is an important point to be made transparent from the development of the inTouch Program and pathways. The inTouch Program success is the result of investment, vision, engagement, and negotiation driven by health professionals focused on patient needs. Linked to this, the pathways success is grounded upon supporting, respecting and upskilling patients and prioritising their decisions about their care plans. A key message from this case study is success is via the bottom-up development and implementation of integrated care strategies. This approach has been critical to the development, ongoing application and perceived value by patients and clinicians.

## Reflections on the development of the inTouch Program

During the development and refinement of the WSLHD inTouch Program there were three interrelated, cascading changes that enabled success. Changes to service norms lead to changes in governance, which enabled the reallocation and commitment of dedicated resources. Within the District, the accepted integrated care approach had been nurse led, consisting of a team of community nurses. They understood the patient population, their risk factors, and the strengths and limitations of their service model. The COVID-19 pandemic acted as a disruptor, necessitating an evolution of the integrated care approach. Rather than using a chronic disease model, an integrated care strategy with a safety and quality lens was adopted, allowing safety outcome data to be utilised. This approach required the expansion of the existing, nurse based integrated care service to include a multidisciplinary health and social care team. This new multidisciplinary team comprised of nursing, medical, allied health, social support, operational and logistical support staff. The expanded service team enabled expansion from a physical, clinical focus, to incorporate the addressing of social issues, such as housing, personal support issues, and basic living needs, including groceries.

The second important change was to governance arrangements which elevated the inTouch service’s ability to interact with the District governance processes to enable more agile decision making. The inTouch Team was part of regular meetings and the sharing of information with external agencies and providers, and the State Government. Computing technology overcame physical distancing barriers, facilitating visible, transparent, communication that enabled real-time question and answers, covering clinical, administrative and support issues.

The third significant change was the substantial investment in resources. Dedicated inTouch Program resources allowed the refinement and simplification of processes and procedures, particularly enabling the onboarding of new staff to be expedited. Clear processes and documentation enabled external providers, including GPs and care providers, to work with the inTouch Team to provide the same level of care regardless of where a patient was physically located. Patient needs and diversity were addressed by focusing on health literacy with the expansion of interpreter services, and patients, carers and consumer information translated into the major languages within the District.

The three interrelated changes resulted in a unified inTouch culture and practice across services, underpinned by flexible thinking and problem solving. This included challenging roles, expectations, duties, and tasks contingent upon flexible staff willing to adapt and work differently. Essentially, people strove to put the patient at the centre of all decisions. A high level of individual and team motivation was further reinforced by the fact that staff lived in the community they provided services for; for them, high quality, integrated care was local and personal.

## Lessons learned

- Achieving integrated care requires care delivery systems that are flexible and adaptable for their specific contexts, conditions and components. The task is to adopt a long-term vision, identifying health care and social needs based on an iterative process that continually integrates practice, policy and knowledge developments and refinements.
- Integration, and a learning health system, is achieved by actively engaged patients, care providers and managers across the care continuum from different organisations. Together they constitute the decision-making team resourced and linked with a common care plan informed by information sharing.
- inTouch is a mechanism to ensure safe, high quality patient care is delivered in the most appropriate environment. The inTouch Integrated Care Framework contains six core elements and four strategic and governance enablers that operationalise and link diverse organisations into a living continuum of care.

## Conclusion

The exploration of the inTouch program and three pathways has detailed how to deliver care for different patient cohorts across health and social care settings. The three care pathways demonstrate the viability and necessity to pursue collaboration, cooperation and care navigation, whilst tailoring services to specific contexts and stakeholder needs. inTouch program aims to ensure safe, high quality patient care is delivered across health and social settings, prioritising the patient needs. The inTouch Integrated Care Framework six core elements are operationalised by four strategic and governance enablers to link diverse organisations into a cohesive continuum of care. Moving forward, research is required to focus on developing robust evaluation methods that assess not only the immediate impact of integrated care programs, such as inTouch, but also their long-term impact, sustainability and scalability.

## Figures and Tables

**Table:** 


ABBREVIATION/TERM	DESCRIPTION

**Care navigation**	A facilitated care coordination process that helps people navigate their way through the health system

**GP**	General Practitioner

**HITH**	Hospital in the Home

**inTouch**	A Program delivering community-based care to people who are at risk of unnecessary emergency department attendance or hospitalisation by enhancing access to appropriate community-based services including general practice, community health, private allied health, and specialist outpatient care

**NSW Health**	The New South Wales Ministry of Health

**Pathway**	The care management processes and requirements, such as assessments, models of care and resources, needed to provide long term care for defined groups of people

**PCBH**	Planned Care for Better Health, an NSW Health Integrated Care Initiative that has been adapted into a Pathway for the inTouch Program

**RACF**	Residential Aged Care Facility

**RACS**	Residential Aged Care Service

**WSLHD**	WSLHD Western Sydney Local Health District

**WSPHN**	Western Sydney Primary Health Network, the Primary Health Network (PHN) for the Western Sydney region of New South Wales

